# Photodynamic therapy vs. photochemical internalization: the surgical margin

**DOI:** 10.1186/1758-3284-3-53

**Published:** 2011-12-22

**Authors:** Waseem Jerjes, Tahwinder Upile, Hani Radhi, Colin Hopper

**Affiliations:** 1 UCL Department of Surgery, University College London, London, UK; 2 Oral and Maxillofacial Surgery Unit, AL-Mustansirya University, Baghdad, Iraq; 3 Leeds Institute of Molecular Medicine, School of Medicine, University of Leeds, Leeds, UK; 4 Chase Farm & Barnet NHS Trust, Enfield, UK; 5 Head & Neck Unit, University College London Hospital, London, UK

## Abstract

Controlling tumour margins in head and neck surgery is of the utmost importance in preventing loco-regional spread and distant metastasis, which will ultimately lead to a significant reduction in morbidity and mortality. We comment on the surgical margins in photodynamic therapy and photochemical internalization.

## Introduction

Several studies assessing diseased tissues in the head and neck region have identified two clinic-pathological parameters, the surgical margin and nodal involvement, which are of utmost importance in assessing tumour-related progression, recurrence, morbidity and mortality [1].

The surgical margin concept has been around for sometime now. It represents the visible margin of macroscopically tumour-free area during surgery or histopathologically proven tumour-free area during processing of the specimen. The status of this margin has been identified to play a vital role in assessing disease status in the head and neck. For example, the presence of non-cohesive invasive front, dysplasia at margin, lympho-vascular invasion, nerve invasion and bone/cartilage may suggest aggressive cancerous growth which is more likely to resist adjuvant therapy and have high recurrence rate [1].

The traditional treatment for any cancer may include 3 modalities: surgery, radiotherapy and chemotherapy. Development in scientific methodology and clinical research has lead to identification of other modalities that were found superior to the traditional ones for specific diseases of certain stage and grade (i.e. hormonal therapy for early prostate cancer), also the identification of other non-traditional adjuvant therapy that could improve the efficacy of other treatments or sometimes act independently (i.e. immunotherapy).

When managing head and neck disease, surgery continue to be the primary intervention with chemo-radiation as adjuvant therapy; but have been applied as neo-adjuvant in certain cases. Keeping an open mind in cancer management is a must as no "magic bullet" has been identified to date and most interventions are equally effective, leading mostly to poor survival rates in most cancers.

Over the last 2 decades, photodynamic therapy (PDT) was introduced as a minimally-invasive surgical modality in cancer management and several studies have proved its usefulness in oncological care [2,3].

### Photodynamic therapy

Using different photo-sensitizers, PDT successfully targeted pathologies in the head and neck, skin, brain, lung, pancreas, intra-peritoneal cavity, breast and prostate. The resultant photochemical reaction (between a photosensitizer, oxygen in the suspect tissue and the light delivered) lasts for few hours and leads to selective injury to the target tissue. All the clinical studies published on this technology showed that PDT is comparable to all the three conventional modalities (i.e. surgery, radiotherapy and chemotherapy) when it comes to disease mortality in the head and neck but identified PDT to be superior as it causes much less morbidity [2,3].

The growing body of evidence regarding PDT's efficacy suggests that it will have a leading role in minimally-invasive surgical oncology. Long-term studies showed that the outcome improves when PDT is offered on a repeatable basis rather than one time only. A recent study on T1/T2 oral cancer showed that 3 rounds of PDT are as successful as surgery in terms of mortality with minimal morbidity. The repeatability is required as residual or recurrent tumour islands have been identified in previously treated PDT-margins (surgical margins), according to the study [4]. It is worth to highlight the fact that most of the cohort included patients with thick T2 disease with moderately to poorly differentiated squamous cell carcinomas. Surgical biopsies identified several parameters indicating aggressive tumour growth.

It is hoped that with the development of third generation photo-sensitizers or advancement in photodynamic applications (i.e. photochemical internalization), a real solution to surgical margins problem in PDT may be found.

### Photochemical internalization

Photochemical internalization (PCI) is a novel technology facilitates the delivery of macromolecules into cytoplasm. It can best be described as sub-lethal PDT facilitating the effect of chemotherapy. The initial mechanism and practical application was described by Berg et al. in 1999 [5]. In 2001, the same group documented the *in vivo *approach to site-specific cancer therapy via photochemical internalization. The outcome of the study revealed synergetic effect of combing photo-activation of photosensitizer and gelonin and the resultant PCI reaction [6]. These findings highlighted the potential clinical usefulness of PCI in cancer therapy, gene therapy and vaccination. In 2005, bleomycin was introduced as the chemotherapeutic agent in PCI [7].

## Discussion

A recent *in vitro *study by Norum et al. [8] had shown that photodynamic therapy (PDT) is less efficient in the tumor periphery than in the tumor center, when compared to photochemical internalization (PCI). In their study, they suggested that PCI may release endocytosed bleomycin into the

cytosol by photochemical rupture of the endocytic vesicles and that PCI caused larger necrotic areas and the regrowth in the peripheral zone was almost completely inhibited after PCI. This study suggested that the PCI effect may result in tumour clearance in the peripheries (surgical margin) of the treated cancerous lesions.

The first PCI trial in humans, using amphinex as the photosensitiser and bleomycin as the chemotherapeutic agent are taking place at University College London Hospital. The trial clinical results, when published, will help shed light on the real effect of PCI on the surgical margins or peripheries of human pathologies.

## Conclusion

Controlling tumour margins is of the utmost importance in preventing loco-regional spread and distant metastasis, which will ultimately lead to a significant reduction in morbidity and mortality.

## Competing interests

Colin Hopper is the principal investigator of the PCI trial on human being carried at University College London Hospital, London. Waseem Jerjes was a sub-investigator of the PCI trial. The other authors declare no competing interests.

## Authors' contributions

All authors have contributed intellectually and to the writing of this manuscript. All authors read and approved the final manuscript.
